# Personalized Immunotherapy in Colorectal Cancers: Where Do We Stand?

**DOI:** 10.3389/fonc.2021.769305

**Published:** 2021-11-23

**Authors:** Li-Feng Hu, Huan-Rong Lan, Dong Huang, Xue-Min Li, Ke-Tao Jin

**Affiliations:** ^1^ Department of Colorectal Surgery, Shaoxing People’s Hospital (Shaoxing Hospital, Zhejiang University School of Medicine), Shaoxing, China; ^2^ Department of Breast and Thyroid Surgery, Affiliated Jinhua Hospital, Zhejiang University School of Medicine, Jinhua, China; ^3^ Department of Colorectal Surgery, Affiliated Jinhua Hospital, Zhejiang University School of Medicine, Jinhua, China; ^4^ Department of Hepatobiliary Surgery, Affiliated Jinhua Hospital, Zhejiang University School of Medicine, Jinhua, China

**Keywords:** colorectal cancer, immunotherapy, personalized medicine, neoantigen, heterogeneity

## Abstract

Colorectal cancer (CRC) is the second leading cause of cancer death in the world. Immunotherapy using monoclonal antibodies, immune-checkpoint inhibitors, adoptive cell therapy, and cancer vaccines has raised great hopes for treating poor prognosis metastatic CRCs that are resistant to the conventional therapies. However, high inter-tumor and intra-tumor heterogeneity hinder the success of immunotherapy in CRC. Patients with a similar tumor phenotype respond differently to the same immunotherapy regimen. Mutation-based classification, molecular subtyping, and immunoscoring of CRCs facilitated the multi-aspect grouping of CRC patients and improved immunotherapy. Personalized immunotherapy using tumor-specific neoantigens provides the opportunity to consider each patient as an independent group deserving of individualized immunotherapy. In the recent decade, the development of sequencing and multi-omics techniques has helped us classify patients more precisely. The expansion of such advanced techniques along with the neoantigen-based immunotherapy could herald a new era in treating heterogeneous tumors such as CRC. In this review article, we provided the latest findings in immunotherapy of CRC. We elaborated on the heterogeneity of CRC patients as a bottleneck of CRC immunotherapy and reviewed the latest advances in personalized immunotherapy to overcome CRC heterogeneity.

## 1 Introduction

Colorectal cancer (CRC) had 1.93 million new patients and caused 935,000 deaths worldwide in 2020, making it the third most common cancer and the second leading cause of cancer death in the world (1). The 5-year survival of non-metastatic CRCs is between 70-90%, which is reduced to 12-14% for patients with metastatic CRC (mCRC) (2, 3). In developed countries, CRC accounts for more than 65% of cancers. Although early diagnosis in these countries has improved patient survival, a quarter of patients are still in the metastatic stage at the time of referral, and about half of low-stages patients progress to mCRC (2, 3). Familial adenomatous polyposis (FAP) and hereditary non-polyposis colon cancer (HNPCC) are among the predisposing factors for CRC, accounting for about 5% of CRC cases (4). The primary treatment for those CRCs that are limited to the colon wall is a combination of surgery, radiotherapy, and chemotherapy based on fluoropyrimidine, irinotecan, and oxaliplatin such as FOLFOX and FOLFIRI (5). The metastatic cases are usually not resectable. The combination of radio/chemotherapy and new targeted therapies against epidermal growth factor receptor (EGFR) and vascular endothelial growth factor (VEGF) are the main treatments in this group (5–7).

CRC is among the cancers with tremendous somatic mutations causing high heterogeneity (8, 9). In 15% of CRCs, the high mutational burden (>12 mutations per megabase) is due to a defect in the genes of mismatch repair (MMR) systems such as MLH1, MSH2, MSH6, and PMS2, which causes transcriptional problems, especially in regions with repetitive nucleotides such as microsatellites. This group has microsatellite instability and is called deficient MMR/microsatellite instability-high (dMMR/MSI-H) CRCs. The second category, which comprises 85% of CRCs, has a much lower mutational burden (<8.24 mutations per megabase) and is known as proficient (p)MMR/MSI-low(L) (10, 11). dMMR/MSI-H CRCs have a better prognosis and survival (15% higher survival rate) than the pMMR/MSI-L group (12). Another cause of high mutational load in CRC is mutations in the catalytic subunit of DNA polymerase epsilon (POLE), which is observed in 3% of CRC patients and causes an ultramutated subtype with more than 100 mutations per megabase (10, 13–15). Approximately 40% of CRC cases have been reported to have mutations in KRAS, which induces cell proliferation and angiogenesis and inhibits apoptosis (5). Over 5,000 different KRAS mutations have been identified in CRC (5). Mutations in MMR, POLE, and KRAS, along with mutations in BRAF, NRAS, and other genes, cause high heterogeneity in the treatment response hindering CRC treatment (16, 17). Therefore, the development of new effective treatment strategies for CRC seems necessary (18).

In recent decades, the remarkable success of immunotherapy in achieving long-lasting responses in solid tumors such as melanoma and lung cancer has led to a strong tendency towards the immunotherapy of other tumors (2, 19). Despite the relative responses of CRC patients to immunotherapy, clinical trials have shown that patients do not respond equally to these treatments, and special attention should be paid to the tumor microenvironment (TME) and patient-related factors in each individual (5). The advances in CRC classification based on the mutational signatures have facilitated the decision-making for appropriate immunotherapy. However, the poor prognosis of mCRC patients indicates the need for more personalization of immunotherapy in CRC patients.

In this article, we provided the latest findings in immunotherapy of CRC. We elaborated on the heterogeneity of CRC patients as a bottleneck of CRC immunotherapy and reviewed the latest advances in personalized immunotherapy to overcome CRC heterogeneity.

## 2 CRC Immunotherapy: Common Approaches

### 2.1 CRC Microenvironment

CRC microenvironment (CRCME) is a heterogeneous microenvironment containing a variety of immune cells. Numerous studies have linked high infiltration of CD8^+^ T cells, CD4^+^ type-1 helper T cells (Th1), follicular helper T cells (Tfh), M1 macrophages, natural killer (NK) cells, and dendritic cells (DCs) with good prognosis in CRC. Contrarily, high infiltration of myeloid-derived suppressor cells (MDSCs), B cells, M2 macrophages, and Th17 cells are associated with poor prognosis (20–23). MDSCs induce pre-metastatic niches, increase angiogenesis, migration, and invasion of tumor cells, and make tumor chemoresistant (24–26). MDSCs also suppress the antitumor activity of NK and T cells and recruit other immunosuppressive cells such as regulatory T (Treg) cells into the tumor site (27, 28). The frequency of MDSCs and Treg cells in the blood and tumor of CRC patients is higher than healthy individuals and healthy tissues around the tumor, respectively (21, 22, 29, 30). However, the relationship between the amount of Treg cells and disease prognosis is still controversial, with some studies associating it with a bad prognosis and some with a good prognosis (31–34).

CRCME is immunologically active in which a variety of immune cells are infiltrated, and different tumor-associated antigens (TAA) and tumor-specific antigens (TSA) are expressed. Besides, numerous molecular markers and receptors such as VEGF, EGFR, insulin-like growth factor-1 receptor (IGF-1R), human epidermal growth factor receptor-2 (HER2), integrins, mucin 5AC (MUC5AC), death receptor-5 (DR5), cytotoxic T-lymphocyte-associated protein-4 (CTLA-4), programmed cell death protein-1 (PD1) are overexpressed in the CRCME (35–43). Hence, immunotherapy targeting these molecules could be a promising therapeutic candidate for CRC treatment (18). Various immunotherapy methods, including monoclonal antibodies (mAbs), immune-checkpoint inhibitors (ICIs), adoptive T cell therapy (ACT), and cancer vaccines, are currently used in CRC (44).

### 2.2 Antibodies and Derivatives

Approval of mAbs such as Bevacizumab, Cetuximab, Panitumumab, and Ramucirumab as the first and second line of treatment with chemotherapy in mCRC led to greater interest in CRC immunotherapy (5, 45). In 2004, two mAbs, Bevacizumab and Cetuximab, were approved for the treatment of mCRC (5, 18). Bevacizumab is a humanized antibody against VEGF that inhibits angiogenesis, and combined with chemotherapy, increases overall survival (OS) and progression-free survival (PFS) in mCRC patients (46). Other anti-angiogenic mAbs include Ramucirumab and Tanibirumab, which are anti-VEGF receptors (VEGFR). The former has been approved as the second line of mCRC therapy combined with chemotherapy, and the latter has not been approved thus far but has shown positive results in reducing tumor growth (47, 48). Vanucizumab is a bispecific antibody against VEGF and angiopoietin-2 that suppresses angiogenesis and metastasis (49). Aflibercept is a fusion protein containing the extracellular domains of VEGFR1,2 fused to Fc of human IgG, which inhibits VEGF and, in combination with chemotherapy, improves the OS of mCRC patients (50).

Another group of mAbs approved for mCRC is anti-EGFR mAbs, including Cetuximab, Panitumumab, and Necitumumab (18). The first two mAbs are confirmed as the first and second line of treatment in mCRC patients with wild-type Ras, respectively (51, 52). Necitumumab has a higher affinity for EGFR than the other two mAbs. It has shown positive results in reducing tumor growth in mCRC patients, which should be approved in further trials (53). Various studies have reported that patients with mutations in KRAS and other RAS genes are resistant to anti-EGFR mAbs, suggesting the need to evaluate RAS mutations before selecting appropriate treatment (5, 54–56). Other mAbs used in mCRC, including anti-DR5 and anti-IGF-1R mAbs, showed transient effects as monotherapy or in combination with chemotherapy (57).

### 2.3 Immune-Checkpoint Inhibitors

In recent decades, ICIs have achieved astonishing success in treating solid tumors that had not previously responded well to treatment (2). ICs physiologically suppress the overreaction of immune cells to prevent autoimmunity. By increasing the expression of ICs, tumor cells misuse their inhibitory signals to escape the antitumor immune responses (58, 59). ICIs block ICs or their ligands on the surface of immune cells or tumor cells to restore the antitumor immune responses (2).

The use of ICIs in unclassified CRC patients did not provide an acceptable overall response. It was observed that some patients responded appropriately to the ICIs while the others were ICI-resistant (60, 61). Further studies revealed that hypermutant tumors express a high level of ICs, leading to appropriate responses to ICIs (62). In fact, in tumors with a high mutational burden, neoantigens are more likely to be produced and presented, and immune cells are more likely to infiltrate hypermutant tumors. That is probably why such tumors have a good prognosis and responses to various immunotherapies (63). CRCME in dMMR/MSI-H tumors is highly infiltrated with CD8^+^ T cells, Th1 cells, macrophages, and enriched with IFN-I (63, 64). Interestingly, the amount of somatic mutations in CRC is associated with treatment response (65). Given the promising results of ICIs in increasing OS and PFS of dMMR/MSI-H patients, the Chinese Society of Clinical Oncology (CSCO) guideline recommends anti-PD1 (Nivolumab and Pembrolizumab) as the first line of treatment for dMMR/MSI-H mCRC patients (66). Besides dMMR/MSI-H patients, POLE-mutated patients also have a high immune cell infiltration and IC expression, resulting in a favorable prognosis and response to ICIs (67).

Unlike hypermutant patients, ICI in pMMR-MSI-L patients did not show promising results, possibly due to the lack of tumor infiltration with immune cells (2, 68). The combination of ICI with radio/chemotherapy and anti-angiogenic agents is currently being investigated in these patients (NCT02563002, NCT03122509). Radio/chemotherapy causes the release of neoantigens through direct damages to the tumor cells (69, 70). The released neoantigens and damage-associated molecular patterns (DAMPs) recruit immune cells into the tumor site, leading to patients’ better response to immunotherapy (69–72). Inhibition of the RAS-AMP activated protein kinase (RAS-AMPK) pathway also increases the tumor infiltration of immune cells and improves the response of pMMR/MSI-L patients to ICI (73–75). Though, it did not show significant changes in the OS of pMMR/MSI-L patients compared to other treatments (73–75).

Bispecific antibodies against TAA and CD3 are another way of increasing the infiltration of immune cells into the CRCME to increase the responses to ICI. Since carcinoembryonic antigen (CEA) is a highly expressed TAA in CRC, CEA-TCB (RG7802) binds to the CEA on the surface of tumor cells and CD3 on the surface of T cells, recruiting more T cells into the tumor site. The combination of CEA-TCB with Atezolizumab (anti-PDL1) was encouraging in pMMR/MSI-L patients, and further investigations are ongoing (NCT02324257, NCT02650713) (76). Targeting other ICs such as T-cell immunoglobulin and mucin domain-containing protein-3 (TIM3), Lymphocyte activation gene-3 protein (LAG3), T cell immunoreceptor with immunoglobulin and ITIM domains (TIGIT), and V-domain immunoglobulin suppressor of T-cell activation (VISTA), as well as the use of agonist for costimulatory molecules such as CD27, OX40, 4-1BB, glucocorticoid-induced tumor necrosis factor receptor (GITR), and CD40 are under investigation in both pMMR and dMMR patients with positive results (2, 64, 77–84).

Besides pMMR/MSI-L patients, the use of ICIs in a large proportion of dMMR/MSI-H patients was not clinically significant (85, 86). For example, tumors with mutations in β2M have defects in the expression of β2-microglobulin with major histocompatibility complex (MHC)-I, leading to antigen presentation deficiency and immune escape of tumors (87). β2M mutation causes resistance to ICI even in dMMR tumors (65, 87). Mutations in genes involved in the antigen processing and presentation pathways as well as in IFN-γ signaling pathways also cause resistance to ICI (64, 88, 89). To sum up, the above cases show that the use of immunotherapy regardless of the tumor and patient conditions leads to treatment inefficiency, which indicates the need to examine each individual’s tumor to select the appropriate treatment option.

## 3 CRC Heterogeneity: A Challenge of Immunotherapy

CRC is one of the cancers with high heterogeneity among patients and even within a tumor, which has challenged the treatment of this disease (6). Genetic and environmental differences, diverse mutations, differences in the infiltration of immune cells into the CRCME, and even differences in the nutrition and microbiome of patients cause extensive heterogeneity in CRC (16, 17, 90–92).

Hypermutation is a hallmark of CRC, although 20% of these mutations cause cancer and far fewer mutations are common in two or more tumors (6). Patients within a mutational group are even heterogeneous (93). For example, in patients with the KRAS mutation, those with the mutation in codon 13 (G13D) respond to the combination of chemotherapy and targeted therapy, while patients with the mutation in codon 12 (G12R) do not respond well (94). Heterogeneity is even associated with the location of the tumor. It has been observed that BRAF mutations are more common in tumors originating from the right side of the body, and these tumors are hypermutant/MSI-H (6, 95). On the other hand, left tumors usually show chromosomal instability and gene expression profiles associated with EGFR pathway activation (13, 96, 97). These differences also influence the choice of treatment options. Generally, anti-EGFR and chemotherapy are prescribed for left-sided tumors, while a combination of Bevacizumab and chemotherapy is recommended for right-sided tumors (5).

In addition to interpersonal heterogeneity, a tumor has heterogeneity within it (98). This heterogeneity can be manifested between cells in a tumor, between metastatic lesions from a tumor, and even between cells in a metastatic lesion (16). Surprisingly, tumors with the same genetic lineage can exhibit different behaviors, growth rates, and treatment responses (99). It has been revealed that increased intra-tumor heterogeneity is directly associated with poor prognosis and decreased OS/PFS in patients (100). Studies of multiple biopsies from various CRC sites have shown that over 65% of tumors have intra-tumor heterogeneity, and there was 10-30% heterogeneity in KRAS and BRAF mutations within tumors (101–104). Heterogeneity of tumors also changes during cancer progression. In the early stages of CRC, intra-tumor heterogeneity is high and decreases during the disease progression (105). Such vast heterogeneity undermines the value of single-biopsy in determining the phenotype and mutational profile of the tumor to select the appropriate treatment. It accentuates the need for further examination of the tumor with biopsy from different areas and the use of advanced instruments to evaluate CRC heterogeneity (101, 106).

### 3.1 Efforts to Overcome CRC Heterogeneity

#### 3.1.1 CRC Classification

One way to overcome CRC heterogeneity, especially in choosing the appropriate treatment strategy, is to classify CRC patients from different molecular and immunological aspects. A successful step in this field was the Consensus Molecular Subgroups (CMS) presented by CRC Subtyping Consortium (13, 107). CMS1 subtype includes MSI tumors with high mutational burden and active anti-tumor responses thanks to high infiltration of anti-tumor cells such as CD8^+^ T cells, Th1 cells, DCs, NK cells, and M1 macrophages as well as a minimum amount of Treg cells (13, 64, 108). CMS1 is known as the MSI-immune subtype and has the best prognosis and well response to immunotherapy (109). The CMS2 subtype is called “canonical”, highlighting its epithelial features and activation of the WNT and MYC pathways. Tumors with the lowest MSI (less than 2%) are in this group (64). Due to the low mutation of these tumors, the infiltration of immune cells is very low, making them known as the immune-desert subtype (6, 13, 108). Given the dysregulated metabolic pathways, CMS3 tumors are called metabolic types. These tumors have mutations in KRAS, and some of them are MSI (13, 107). Immune cell infiltration in CMS3 is slightly higher than CMS2 but still low and has an immunologically-inactive CRCME, referred to as immune-excluded subtype (13, 64, 107). Eventually, CMS4 tumors are called mesenchymal type because they have mesenchymal properties such as strong endothelial-mesenchymal transition activity and high stromal content (13, 107). They are highly infiltrated with immunosuppressive cells such as Treg cells, M2 macrophages, and myeloid cells, and the presence of anti-tumor immune cells such as DCs, activated NK cells, Th1, and CD8^+^ T cells in their CRCME is very low. Besides, CMS4 tumors have activated VEGF, TGF-β, and CXCL12 signaling pathways, all of which cause the worst prognosis of CMS4 among the four categories (64, 108).

#### 3.1.2 Immunoscore

Besides molecular classification, CRCs can also be classified based on immunological properties. As mentioned, the mutational burden is a hallmark for the response to immunotherapy. However, the resistance of some dMMR tumors and the appropriate response of some pMMR tumors suggest that a high mutational burden alone is not responsible for the response to immunotherapy (2). It seems that the mutations indirectly increase the response to immunotherapy by increasing the probability of immune cell infiltration into the CRCME (2). The amount of mutations in dMMR tumors is roughly 20 times more than that of pMMR tumors, which produce more (20-fold) neoantigens, increase immune cell infiltration, and induce antitumor responses (110). As a result, it makes more sense to evaluate the immune status of CRCME to predict the response to immunotherapy. Accordingly, recent studies suggest using immunoscore in predicting the response to immunotherapy (2, 64, 111). Immunoscore is evaluated based on the rate of T cells, especially CD8^+^ T cells in the tumor center and invasive margins of the tumor. Therefore, the high amount of CD3^+^ cells and CD8^+^ cells in both center and margin of the tumor are determined with a score of 4, and the low number of these cells in both areas is determined with a score of zero (111–113). CD45RO has also been used as a marker in some immunoscore assessments (112, 114).

Interestingly enough, stage I patients with low immunoscore have been shown to have a poor prognosis and low rate of disease-free survival (DFS) similar to those in stage IV (114). On the other hand, high immunoscore was observed in some pMMR-MSI-L patients, and the rate of DFS, OS, and recurrence was similar in MSI-H and MSI-L patients with high immunoscore (115). Also, in patients with low immunoscore, microsatellite status had no beneficial effect on survival (115). These observations indicate that the prognostic value of immunoscore in clinical outcome and response to immunotherapy is higher than the conventional classification system (UICC-TNM) and even classification based on MMR and MSI (64, 115). Noteworthy, classifying patients into only four groups based on the presence or absence of a few subtypes of immune cells does not seem sufficient. More advanced immunophenotyping methods are needed by considering other immune cells, the infiltration site of each immune cell subset, immune activation/exhaustion markers, and other immunological parameters.

#### 3.1.3 Heterogeneity Assessment Methods

Despite the efforts to study CRC heterogeneity, our current knowledge in this field is just the tip of the iceberg. Expanding next-generation sequencing (NGS), single-cell sequencing, and whole-exome sequencing techniques along with the application of omics data at various levels, including genomics, epigenomics, transcriptomics, peptidomics, proteinomics, and metabolomics, could give us valuable information on the heterogeneity of CRCs (116–123).

Advances in computational biology tools and integrating information obtained from different omics methods could develop accurate and rapid models to predict tumor behavior and response to treatment (124–126). Examples of attempts to create virtual tumor models include virtual patient-ModCell and genome-scale metabolic models used in various cancers, including CRC (127–129). The purpose of these models is to use literature to obtain predictive and prognostic biomarkers, including molecular, metabolic, and immunological signatures, to predict disease progression and treatment response (124, 130, 131). Therefore, identifying reliable biomarkers is an urgent need to increase the accuracy of these models. This modeling is in its infancy and requires to address challenges such as a large amount of information obtained from different omics for each individual, the precise classification of each patient uniquely into a category, the inclusion of immunological variables in models, and prediction of tumor response to treatment (124).

Due to the tumor’s dynamic behavior, the study of tumor behavior requires the evaluation of accessible biomarkers that can reflect tumor changes during treatment without the need for biopsy. Therefore, identifying biomarkers in body fluids that can accurately, quickly, and cost-effectively reflect the stage and characteristics of the tumor is desired. Circulating exosomes, microRNAs, tumor cells (CTC), tumor DNA (ctDNA) can be ideal indicators for tumor heterogeneity changes in the course of treatment (6). The similarity of the genetic profile of CTCs with tumors has been reported 50-77% (132, 133). Interestingly, the genetic profile similarity between cell-free DNAs (cfDNA) or ctDNAs and tumors has been reported to be more than 90% (134–136). Examination of the genetic contents of exosomes is an available method to study tumor mutations with acceptable accuracy. Surprisingly, KRAS mutant genes are more likely to be loaded in exosomes than normal KRAS genes (137, 138).

An emerging method for modeling patient tumors to evaluate the treatment response is establishing patient-derived organoids (PDOs) (139). PDOs are three-dimensional structures of the extracellular matrix containing patient-derived tumor cells fed with culture medium and growth factors. The close similarity (70-90%) of PDOs with the primary tumor in terms of structure, function, and even heterogeneity has made PDOs more reliable models than cell lines that have only 10% of the characteristics of primary tumors (139–144). PDOs have been shown to predict treatment responses with an accuracy of 88-100% (139–144). However, the absence of immune cells in PDOs limits this model in predicting the response to immunotherapy (64). Recently, air-liquid interface culture systems enable PDOs to infiltrate with different immune cells and fibroblasts, making PDOs very similar to the tumor microenvironment (TME). These models are reliable tools for assessing the response of patients to immunotherapies (145). Further studies are required to develop these models and confirm their ability to predict the response to various immunotherapies in CRC.

## 4 Personalized Immunotherapy in CRC

### 4.1 Cancer Vaccines

Cancer vaccines are immune system boosters that invigorate patients’ immune responses against cancer by exposing tumor antigens to immune cells (44, 146). The immunotherapies discussed so far is targeting TAAs that are overexpressed in the CRCME. Most TAAs targeted in CRC include CEA, EGFR, VEGFR1/2, survivin, mucin-1 (MUC-1), melanoma antigen gene (MAGE), Wilms tumor antigen-1 (WT1), transmembrane-4 superfamily member-5 protein (TM4SF5), mitotic centromere-associated kinesin (MCAK), ring-finger protein-43 (RNF43), translocase of the outer mitochondrial membrane-34 (TOMM34), squamous cell carcinoma antigen recognized by T cells-3 (SART3), insulin-like growth factor II m-RNA-binding protein-3 (IMP3), kinase of the outer chloroplast membrane-1 (KOC1), 5T4, guanylyl cyclase C (GUCY2C), and human telomerase reverse transcriptase (hTERT) (44, 147–160).

Another type of cancer antigen is TSAs or neoantigens, which are nucleotide or polypeptide sequences that are mutated and might be identified as non-self antigens (44). Neoantigens are expressed only on the surface of tumor cells and significantly reduce the risk of adverse effects of vaccination (15, 161). Hypermutant tumors have a higher chance of producing neoantigens, which is one reason for the increase in immune cell infiltration and good prognosis of these tumors (110, 162). The specificity of neoantigens and their association with the prognosis have made neoantigens ideal targets for cancer immunotherapy, especially for personalized cancer vaccines (64). Neoantigens are generated from frameshift and framework mutations, single nucleotide or structural variations, insertions or deletion, and alternative splicing (146). Most of the neoantigens produced in CRC are derived from TTN gene mutations. Mutations in other genes such as TGFBR2, TAF1B, HT001, MARCKS1/2, CDX2, AIM2, TCF7L2, KRAS, PCNXL2, BAX-1, MUC16, SOX9, RNF43, KMT2D, ARID1A, APC, ZFP5N2 could also produce neoantigens (146). Mutations that occur in tumors are divided into driver and passenger types. The products of driver mutations are critical for the survival of tumor cells, and therefore targeting these mutations reduces the likelihood of immune escape by the tumor (15).

Vaccination with short peptides induces a limited immune response. Therefore, the cocktail of several long peptides is usually used in vaccines to induce both class-I and class-II MHC-mediated responses (150, 163, 164). Frameshift mutations generate long neoantigenic peptides with predictable sequences that can be used in vaccines. Interestingly, the number of vaccine peptides that induce the CD8^+^ T cell responses is directly related to patient survival (151). It has been demonstrated that vaccines with more than two neoantigens were able to control the tumor for a long time in preclinical and clinical models (165). In a trial, vaccination with three frameshift-derived neoantigens was performed on advanced-stage CRC patients, which induced a response in all patients and stopped the disease in one patient (166). Due to the high heterogeneity of CRC patients, some studies are attempting to use cocktail neoantigens or establish a library of DNA, RNA, and peptide vaccines as off-the-shelf vaccines (150, 151, 167–169). The list of clinical trials in neoantigen-based off-the-shelf vaccines and ACT is provided in **Table 1**.

**Table 1:** Clinical trials on CRC immunotherapy targeting shared neoantigens.

Type of immunotherapy	Title	Phase	No. patients	Combination	Status/outcome	Ref./CT code
**Neoantigen Vaccine**	Mutant KRAS-targeted long peptide vaccine	I	30	Nivolumab and ipilimumap	Active	NCT04117087
	13mer Mutant KRAS neoantigens	II	38	IL-2 and GM-CSF	- 4 patients had stable disease	(170)
	(Class I and II peptides)				- 34 patients had progressive disease	NCT00019331
					- Peptide-specific T cell response: 54.1%	
					- MST: 16.9m	
					- PFS: 3.6 m	
	13mer Mutant KRAS neoantigens (Class I peptides)	II	7	Detox (adjuvant)	- 4 patients remained with no evidence of disease	(171)
					- More than two times increase in IFN-γ	
					- DFS:27.2m	
					- OS: 41.5m	
	Frameshift peptides Neoantigens: AIM2 (-1), HT001(-1), TAF1B(-1)	I/II	22	Montanide ISA-51 VG	- 16 patients showed immune response (CTL/IgG induction)	(172)
						NCT01461148
	Frameshift-derived neoantigen-loaded	I/II	25	–	Active	NCT01885702
	DC vaccine					
**Adoptive cell therapy**	Anti-KRAS G12V Engineered T cells	I/II	110	Cyclophosphamide, Fludarabine, Aldesleukin	Active	NCT03190941
	TCR-engineered T cells against	I/II	1	–	Terminated	NCT03431311
	TGFβRII frameshift peptide					

CT, Clinical trial; IL-2, Interleukin-2; GM-CSF, Granulocye-macrophage colony-stimulating factor; MST, Median-survival time; PFS, Progression-free survival; DFS, Disease-free survival; OS, Overall survival; IFN, Interferon; DC, Dendritic cell; TCR, T cell receptor; TGFβRII, Tumor growth factor β receptor II.

Neoantigen vaccines are extensively investigated in melanoma and glioblastoma with promising results in increasing patients’ survival (173–175). Given that glioblastoma has a low mutational and neoantigenic burden (8, 176, 177), these results suggest that neoantigen-based vaccines are effective even in tumors with low mutation rates such as pMMR/MSI-L (64, 175). Neoantigen vaccines may improve the infiltration of immune cells into CRCME and increase the immunoscore of tumors, leading to increased tumor responses to ICIs and other immunotherapies (64). The combination of neoantigen vaccines with ICI is currently being investigated in clinical trials (NCT02600949, NCT03289962).

In neoantigen vaccines, prioritizing the selection of neoantigens is a critical step influencing the effectiveness of vaccination. Some studies prioritize selecting those with a high prevalence in CRC patients (146, 167, 168). However, in personalized immunotherapy, the goal is to identify the neoantigens expressed in the patient’s tumor and select the most immunogenic and appropriate ones for vaccination. The application of the NGS technique to perform whole-exome sequencing, whole-genome sequencing, and RNA sequencing, together with the use of omics data, helps us identify mutated regions and neoantigens created in the patient tumor to be used in the personalized vaccine (146).

**Figure 1:**
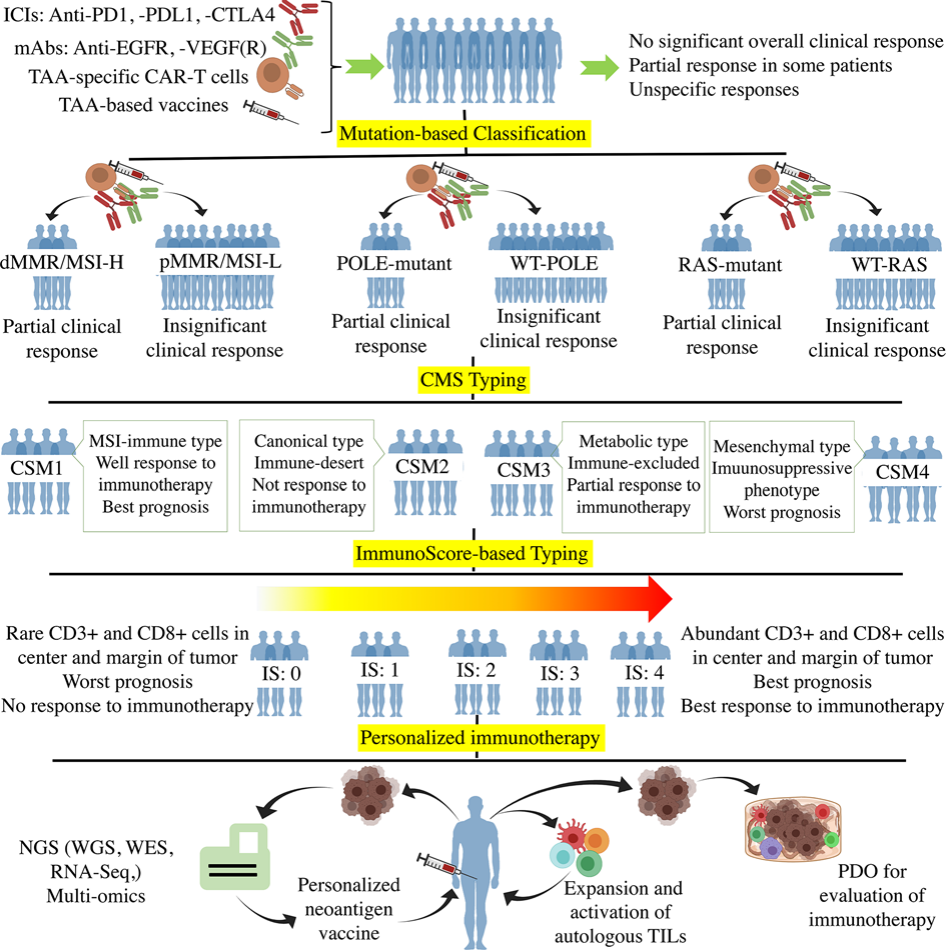
The Process of personalized immunotherapy in CRC. Off-the-shelf immunotherapy such as ICIs, mAbs (anti-EGFR, -VEGF(R)), TAA-specific CAR-T cells, and TAA-base cancer vaccines resulted in tumor regression and stable disease in some patients. However, such immunotherapy did not achieve significant overall clinical responses when used in unclassified CRC patients. The mutation-based classifications according to MMR, POLE, and RAS genes showed that the mutant subtypes are more responsive to immunotherapy. CMS typing more clarified the CRC classification using molecular differences. Regarding the fact that immunotherapy is the method of choice for those tumors with high infiltration of immune cells, the immune-based classification such as immunoscore (Ranged between 0-4) grouped the patients based on the tumor infiltration of immune cells. Ultimately, Personalized immunotherapy tried to classify each patient as an independent group in which the individualized neoantigens is determined through the hi-tech NGS and multi-omics to optimize and personalize the cancer vaccines. Besides, autologous ACT and PDO-based prediction of the immunotherapy responses are the other approaches towards personalized immunotherapy. ICI, Immune-checkpoint inhibitor; PD1, Programmed cell death protein 1; PDL1, PD ligand 1; CTLA4, cytotoxic T-lymphocyte-associated protein 4; TAA, Tumor-associated antigen; EGFR, Epidermal growth factor receptor; VEGFR, Vascular-endothelial growth factor receptor; CAR-T cell, Chimeric antigen receptor T cell; d/pMMR, Deficient/proficient miss match repair; MSI-H/L, Microsatellite instability high/low; POLE, DNA polymerase epsilon; CMS, Consensus molecular subgroup; NGS, Next-generation sequencing; WES, Whole-exome sequencing; RNA-Seq, RNA sequencing; TIL, Tumor-infiltrating lymphocyte; PDO, Patient-derived organoid.

Due to the presentation of all antigens in the body by MHCs, determining patient MHC profile and selecting appropriate peptides with high binding affinity to the patient MHCs should also be considered to select immunogenic neoantigens (146). Many personalized vaccine studies consider the presence of targeted neoantigens in the patient’s tumor as a criterion for vaccine design. The next step in achieving an effective vaccine is to assess the patient’s immune status in terms of the presence or absence of previous immune response to the selected neoantigen (178, 179). Tumor-derived neoantigens that have not been able to stimulate the immune responses cannot induce strong immune responses in the vaccine formulation (180, 181). Therefore, after selecting the ideal neoantigens and matching them with the patient MHC, it is necessary to evaluate the pre-existing immune responses against those neoantigens by determining the frequency of antigen-specific cytotoxic T cells (CTLs) as well as the antigen-specific IgG titer in the serum (15, 182). Considering the pre-existing immunity in neoantigens selection could enhance the vaccination efficacy and reduce the adverse events (15). Clinical trials using this type of personalized vaccination have shown encouraging results in inducing an antitumor immune response and increasing OS even in chemoresistant CRC patients (178, 179, 182). The process of personalized immunotherapy in CRC is illustrated in **Figure 1**.

Neoantigen vaccines can be designed and produced in various forms. Peptide/protein vaccines are safe and have a convenient and cost-effective production process but usually do not have sufficient immunogenicity and require adjuvant (178, 183, 184). In addition, the antigenic escape necessitates the need to use antigen cocktails (151, 178, 184). The AutoSynVax vaccine is a personalized vaccine using neoantigens and QS-21 adjuvant that is undergoing a phase 1 clinical trial in advanced cancers (NCT02992977).

DNA and mRNA vaccines are highly immunogenic due to the presentation of produced antigens by both MHC-I and -II pathways. They also stimulate the innate immune sensors in the cytosol (185–187). The mRNA vaccines are safer than DNA vaccines due to the lack of concerns about unwilling integration to the host genome (44, 188). Also, the convenience, speed, and inexpensiveness of mRNA manipulation have led to the widespread acceptance of mRNA vaccines in personalized immunotherapies, which has yielded promising results in CRC (188–190). DCs are ideal candidates to carry the neoantigens into the tumor because they can present neoantigens *via* both MHC-I/-II pathways and provide costimulatory molecules required for optimum immune responses. They can be pulsed *ex vivo* with neoantigens or mRNA, matured with cytokines, and then returned to the patient as autologous DC vaccines (191). These vaccines are being tested in clinical trials (**Table 2**).

**Table 2:** Clinical trials on personalized immunotherapy of CRC.

Type of immunotherapy	Title	Phase	No. patients	Combination	Status/outcome	Ref./CT code
**Peptide-based neoantigen vaccines**	Personalized neoantigen-based peptide vaccine	I	10	TS-1	- Partial response: 10%	(192)
					- Peptide-specific immune response: 50%	
	Personalized neoantigen-based peptide vaccine	I/II	11	UFT, UZEL	-Stable disease: 36%	(193)
					-Peptide-specific cellular and humoral response: 64 and 82%, respectively	
	Personalized neoantigen-based peptide vaccine	I	13	–	-Stable disease: 43%	(194)
					- Peptide-specific cellular and humoral response: 90 and 80%, respectively	
					-MST: 19.6 months	
	Personalized neoantigen-based peptide vaccine	II	60	Chemotherapy	- Peptide-specific cellular and humoral response: 63 and 49%, respectively	(195)
					-MST: 16.6 m	
					-1-year survival rate: 53%	
					2-year survival rate: 22%	
	Personalized RAS Mutant neoantigens	I	10	Detox (adjuant)	-Stable disease: 10%	(196)
					-Neoantigen-specific CD4^+^/CD8^+^ T cell responses: 30%	
	Personalized Synthetic peptide Vaccine	1	60	Imiquimod, Pembrolizumab	Active	NCT02600949
	Personalized neoantigen peptide vaccine	I	12	Poly-ICLC adjuvant	Active	NCT04799431
				Retifanlimab		
				(anti-PD1)		
	Personalized neoantigen cancer vaccine	I/II	214	Nivolumab and Ipilimumab	Active	NCT03639714
	(GRT-C901 and GRT-R902)					
	Patient-specific neoantigen cancer vaccine using NGS and HLA typing	Observational	93	–	Completed-Results pending	NCT03794128
	Individualized peptide-based immunization	Observational	100	–	Active	NCT03871790
	Personalized neoantigen cancer vaccine (GRT-C903 and GRT-R904)	I/II	144	Nivolumab and Ipilimumab	Active	NCT03953235
**mRNA-based neoantigen vaccines**	RO7198457 (mRNA-based	I	567	Atezolizumab	Active	NCT03289962
	Individualized neoantigen vaccine)					
	mRNA-based personalized vaccine targeting neoantigens	I/II	5	–	-The vaccine was safe and induced the neoepitope-specific T cell responses	(197)
					-No objective clinical response was observed	NCT03480152
**Vector-based neoantigen vaccines**	(ADXS-NEO) Listeria monocytogenes-based personalized tumor neoantigens vaccine	I	5	With or without Pembrolizumab	Active	NCT03265080
	Personalized neoantigen yeast-based vaccine, YE-NEO-001	I	16	–	Active	NCT03552718
	Personalized live, attenuated, double-deleted Listeria monocytogenes (pLADD)-based vaccination with TSA	I	28	–	Terminated	NCT03189030
**Autologous tumor cells/lysate**	OncoVax (autologous tumor cell)	III	550	Surgery	Active	NCT02448173
	Autologous tumor lysate	I/II	27	CIK	-Serum level of IFN-γ and IL-	(198)
					12 raised	
					-Five-year DFS rate: 66%	
	Autologous or allogeneic whole tumor cell	I/II	50	–	Active	NCT00722228
	Autologous or allogeneic tumor cell vaccine	II	40	IFN-α	Completed- Results pending	NCT00002475
				IFN-γ		
				Sargramostim		
				Cyclophosphamide		
	Autologous tumor cell vaccine plus BCG vaccine	I/II	30	BCG vaccine	Completed-results pending	NCT00016133
				Fluorouracil		
				Leucovorin Calcium		
				Adjuvant therapy		
	Autologous tumor cell vaccine plus BCG vaccine	III	412	BCG vaccine	-Development of immune response in vaccinated patients results in insignificantly improved DFS and OS	(199)
	Autologous tumor cell vaccine plus BCG vaccine	III	254	BCG vaccine	-44% decrease in recurrence	(200)
					-Improved relapse-free survival	
	Autologous tumor cells infected with Newcastle disease virus	III	50	–	-Insignificantly beneficial in increasing overall and metastasis-free survival	(201)
					-could not prevent from recurrent metastases	
**DC-based autologous vaccines**	Autologous tumor-loaded DCs	I	6	–	-Induction of T cell responses against tumor	(202)
	Autologous tumor lysate-loaded DCs	I/II	17	Capecitabine	-Induction of specific anti-tumor responses	(203)
					-Clinical responses: 88%	
					-six months survival: 82%	
	DC pulsed with Autologous tumor lysate	I/II	26	CD40L	-Tumor-specific T cell response: 63%	(204)
					-Improved relapse-free survival	
					-No further responses following CD40L- based activation	
	DC pulsed with autologous tumor RNA	I	1	–	-Induction of tumor-specific CTLs capable of recognizing and lysing autologous, primary tumor cells *ex vivo*	(205)
	Autologous tumor lysates pulsed human dendritic cells vaccine	III	480	Chemotherapy	Active	NCT02503150
	Personalized autologous TSA-DC vaccine	N/A	20	Cyclophosphamide	Active	NCT03185429
	DC vaccine pulsed with autologous tumor proteins plus autologous tumor infiltrating lymphocyte	II	70	Aldesleukin	Completed-results pending	NCT00019084
				Sargramostim		
	Autologous DC vaccine with autologous tumor antigens	II	52	–	Completed-results pending	NCT01413295
	Autologous Dendritic Cell Vaccine	I/II	28	Avelumab	Completed-results pending	NCT03152565
	Personalized neoantigen-primed DC vaccine	I	80	–	Active	NCT04147078
	Autologous DC pulsed with tumor lysate	II	19	IL-2	Active	NCT02919644
	Autologous DC pulsed with tumor antigens	II	58	–	Active	NCT01348256
	Autologous DC pulsed with tumor lysate antigens	I	30	–	Active	NCT03214939
**Adoptive Cell therapy**	Personalized neoantigen-targeting CD8^+^T cells	I	1	Pembrolizumab	Terminated	NCT02757391
	Gene-edited autologous neoantigen-targeting T cells	I	148	With or without Nivolumab	Active	NCT03970382
				+ IL-2		
	Autologus activated T and NK cells	I/II	86	–	Active	NCT00854971

CT, Clinical trial; TS-1, Tegafur/gimeracil/oteracil; UFT, Uracil-tegafur; UZEL, UFT and leucovorin; MST, Median-survival time; PD-L1, Programmed death receptor-ligand 1; TSA, Tumor-specific antigen; IFN, Interferon; IL, Interleukin; DFS, Disease-free survival; BCG, Bacillus Calmette–Guérin; OS, Overall survival; DC, Dendritic cell; CTL, Cytotoxic T lymphocyte; NK cell, Natural killer cell.

Viral, bacterial, and yeast vectors can also be loaded with genes encoding neoantigens and induce robust immune responses against the tumor (44). These vectors release pathogen-associated molecular patterns (PAMPs) that stimulate the innate and adaptive immune response and increase the infiltration of immune cells into the tumor site (206–210). Adenoviruses, lentiviruses, retroviruses, and Poxviruses are important viral vectors in vaccine design (211). Clinical trials with viral vectors loaded with neoantigens and TAAs have been performed on mCRC patients with promising results in inducing the immune responses and improving OS (212, 213). However, viral vector safety concerns such as the possibility of mutation and becoming a pathogen still remain and need further studies (214, 215). The use of engineered bacteria as vectors of neoantigens in clinical trials has also yielded positive results. ADXS-NEO is a personalized neoantigen vaccine with Listeria monocytogenes vector that increased CD8^+^ T cell infiltration in CRCME in 40% of patients and induced specific responses against 90% of neoantigens (NCT03265080). Yeast vectors are a promising future option for cancer vaccines due to their high safety, ease of large-scale production, no need for adjuvants, and induction of effective responses against neoantigens (207, 216). Several clinical trials are investigating the effectiveness of yeast vectors in mCRC vaccines (44).

Another strategy for personalized vaccination is the use of autologous tumor cells or tumor lysates. Vaccination with whole tumor lysate reduces the possibility of immune escape due to the presence of all tumor antigens (44). However, many of these antigens are normal self-antigens that do not stimulate the immune response and reduce the effectiveness of whole lysate vaccines. Strategies such as using oncolytic viruses *in vivo* and *in vitro* or tumor cells infected with oncolytic viruses release PAMPs, which increases immune responses (217, 218). The results of phase I and II trials showed that the use of Newcastle virus-infected tumor cells reduced recurrence and increased OS in CRC patients (44, 219). However, the high levels of self-antigens present in tumor lysates cause the lack of specificity of immune responses and increase the possibility of promoting autoimmune responses, limiting the use of this method in susceptible individuals (220, 221).

### 4.2 Adoptive T Cell Therapy

ACT is a cancer immunotherapy method in which T cells are collected from the tumor, lymph nodes, or peripheral blood of a patient and returned to the patient’s body after proliferation and selection of tumor-specific T cells. ACT can be performed with unmanipulated cells or engineered cells that express chimeric antigen receptors (CAR-T cells). CAR-T cells are independent of MHCs, and due to carrying costimulatory domains, they could induce strong antitumor responses (2). CAR-T cells are mainly against TAAs that overexpress in CRC, including CEA, EGFR, mesothelin, MUC-1, NKG2D ligand, HER2, c-met, CD133, GUCY2C, epithelial cell adhesion molecule (EpCAM), and Tumor-associated glycoprotein (TAG)-72 (18, 157). These CAR-T cells contain immune activating domains of CD28 and CD137. In the context of mCRC, CAR-T cells as monotherapy or in combination with cytokines such as IL-12 had encouraging effects such as tumor reduction and long-term disease stability in some patients (222–225). However, challenges such as on-target/off-tumor toxicity and damage to other organs due to the lack of specificity of target antigens are seen. Identification of TSAs is one of the current challenges in CAR-T cell therapy (18). A second concern is cytokine release syndrome due to the CAR-T cells activation following binding to antigens in both tumor cells and normal cells (226).

The use of tumor-specific unmanipulated cells has also yielded positive results in CRC. In one study, tumor-infiltrating lymphocytes (TILs) were collected from metastatic lesions of a patient carrying KRAS-G12D mutation. The mutation-specific CD8^+^ T cells were selected and returned to the patient, resulting in the elimination of 85% of metastatic lesions (227). In another study, sentinel lymph node T was used instead of TILs, which resulted in complete response and disease stability in some patients and partial response in others (228, 229). The combination of ACT with chemotherapy and bevacizumab caused 80% overall response, 26.7% complete response, and stopped tumor progression in stage IV CRC patients (230). Various trials are investigating the effects of ACT alone or combined with other immunotherapies such as ICI in CRC patients (NCT03935893, NCT02757391, NCT01174121, NCT03904537). In these trials, various omics data are used to identify personalized antigens (146). These primary successes suggest that ACT, along with neoantigen vaccines, could be promising candidates for personalized immunotherapy in CRC.

### 4.3 Role of the Microbiome in Personalized Immunotherapy

Human microbiome is defined as all the microbiota in and on the human body plus their genomes, structural elements, and productions (231). The microbiota composition depends on genetic and environmental factors such as diet, exposure to chemicals, drugs, especially antibiotics, and is unique to each individual (232). Gut microbiota plays an essential role in the formation and regulation of gastrointestinal immune responses by producing various metabolites (233). Thus, dysbiosis in gut microbiota composition alters their metabolites such as short-chain fatty acids SCFAs, polyphenols, tryptophan catabolites, and some vitamins, and is associated with the pathogenesis of many diseases, including CRC (234). The researchers found several pathogen-derived proteins in the gastrointestinal tract of CRC patients (234, 235). They also showed that the high levels of antibodies against these proteins might have a diagnostic value for CRC patients (235, 236).

Besides, microbiota composition plays a critical role in patients’ treatment responses (237, 238). Presumably, the microbiota diversity among patients is one of the reasons for interpatient variety in response to cancer treatment (238). Microbiota can affect the response to chemotherapy. It has been observed that some bacteria metabolize and reduce the effects of chemotherapy at the tumor site (239). Besides, microbiota are chief players in regulating inflammatory responses in GI, through which they can influence the response of CRC patients to immunotherapy (240–242). Evidence suggests that exposure to antibiotics during anti-PD1 treatment alters the patients’ responses to anti-PD1 (241). Comparing the microbiota of patients who were well-responsive to anti-PD1 with those of patients who were poor-responsive to anti-PD1 showed that the presence of some bacteria (such as Akkermansia, Faecalibacterium, and Bifidobacterium) was associated with a better response to immunotherapy (238, 241). Contrarily, high levels of bacteria such as Bacteroidale are associated with a low response to anti-PD1 therapy (238, 241). This finding indicates that disruption of the microbiota network and reduction of beneficial bacteria reduce the patients’ responses to immunotherapy.

Interestingly, by manipulating the microbiota composition, responses to immunotherapy can be improved. Fecal microbiota transplant (FMT) is a well-known way to manipulate the network of GI microbiota. In FMT, the stool of patients who have responded well to treatment is transferred to the GI tract of non-responsive patients. Chen 2019 Matson 2021 Zhou 2021. It was observed that FMT of mice from anti-PD1-sensitive patients induced a favorable response to anti-PD1 in transplanted mice (241, 243). On the other hand, mice that received FMT from anti-PD1-resistant patients were still resistant to anti-PD1 after transplantation (241, 243). The use of microbiota in improving treatment response has entered clinical studies, and several clinical trials are investigating the combination of FMT with chemotherapy and immunotherapy (NCT04130763, NCT03782428). These studies, which are still in early phases, are investigating the role of FMT or probiotic supplements along with ICIs in CRC patients who have not previously responded well to immunotherapy.

In a nutshell, the evidence suggests that the microbiome is a determinant variable in immunotherapy and is involved in all aspects of CRC, including the diagnosis, prognosis, monitoring, and response to immunotherapy. Therefore, microbiomics should be used along with other omics data in optimizing personalized immunotherapy (238). In this era, molecular pathological epidemiology (MPE) has been recently developed to investigate the relationship between gut microbiota, host, environmental factors, diet, disease etiology and pathogenesis to investigate the role of microbiota and its factors in disease development and treatment response (244–246).

### 4.4 Combination Therapy

Although personalized immunotherapy approaches promise a new horizon in the treatment of CRC, longtime challenges of researchers with cancer treatment show that cancer is the most complex disease that is not supposed to cure with monotherapy (247). Regarding the high heterogeneity of CRC, it seems that it could escape even from personalized immunotherapy (44). Therefore, multi-aspect combinations of immunotherapy, chemotherapy, radiotherapy and other targeted therapies are required to fight cancers. It has been revealed that the combination of immunotherapies such as ICIs and mAbs with chemotherapy and radiotherapy could reinvigorate the antitumor responses and increase the patients’ survival (247). Personalized immunotherapy could also be combined with other therapeutic approaches to maximize the antitumor effects. In this regard, combination therapy using cancer vaccines (peptide, DNA, RNA, DC, and viral vector vaccines), adoptive cell therapy, ICIs, cytokines, chemotherapy, and radiotherapy increased antitumor effects leading to improved overall survival (151, 213, 227, 247–255). A phase I clinical trial of a neoantigen-encoding mRNA cancer vaccine combined with PD-1 blocking showed acceptable safety and well induction of neoantigen-specific immunity leading to partial and complete response in MSI-H CRC patients (189).

Interestingly, combination therapy is also better to be personalized to achieve the optimum response. The systems biology and simulation models can help find the best combination therapy in each patient. More recently, a quantitative systems pharmacology model predicted the efficacy of monotherapy with a bispecific T cell engager, a PD-L1 blocker, and combination therapy in CRC patients based on their individual characteristics (256). The development of these models could offer the best combination therapy for each patient to improve clinical efficacy.

## 5 Conclusion

The high heterogeneity in cancer patients has led to a paradigm shift that considers cancer as an individual rather than a general disease. Significant advances in immunotherapy have raised great hopes for the treatment of poor prognosis cancers. However, different responses of patients to immunotherapy necessitate the classification of patients into smaller groups so that eventually, each patient is in a separate classification and deserving personalized treatment. Personalized immunotherapy has various aspects generally based on specific neoantigens created in each individual’s tumor. Neoantigen-based personalized immunotherapy is now a time-consuming and costly method due to the need for up-to-date tools to identify and prioritize neoantigens. With the advancement of technologies such as sequencing and multi-omics, this type of treatment will be available quickly and affordably soon and will replace the current treatments.

## Author Contributions

Both L-FH and H-RL equally contributed to writing the manuscript and sourcing references for the review. DH contributed to discussions and editing of the manuscript. X-ML and K-TJ conceived the outline of this paper and participated in critical review and further revision of the manuscript. All authors contributed to critical discussions and finalizing the manuscript before submission. They have all given approval to the final form of the manuscript.

## Funding

This work was supported by Zhejiang Provincial Science and Technology Projects (grant no. LGD19H160001 to K-TJ).

## Conflict of Interest

The authors declare that the research was conducted in the absence of any commercial or financial relationships that could be construed as a potential conflict of interest.

## Publisher’s Note

All claims expressed in this article are solely those of the authors and do not necessarily represent those of their affiliated organizations, or those of the publisher, the editors and the reviewers. Any product that may be evaluated in this article, or claim that may be made by its manufacturer, is not guaranteed or endorsed by the publisher.
